# Technical overview and clinical outcomes of transabdominal strategies to eliminate or neutralize staple-line intersection during low anterior resection: a systematic review

**DOI:** 10.1007/s00384-026-05205-8

**Published:** 2026-07-25

**Authors:** Alfredo Annicchiarico, Gianpiero Gravante, Rossella Melcarne, Leonardo Vincenti, Arcangelo Picciariello

**Affiliations:** 1 Department of Surgery, Azienda Sanitaria Locale Di Parma, Vaio Hospital, Fidenza, Italy; 2 Department of General Surgery, Azienda Sanitaria Locale ASL Lecce, Casarano, Italy; 3 Department of Translational and Precision Medicine, Sapienza University of Rome, Rome, Italy; 4 IRCCS de Bellis, Castellana Grotte, 70013 Bari, Italy; 5 Department of Experimental Medicine, University of Salento, Lecce, Italy

**Keywords:** Rectal surgery, Colorectal anastomosis, Anastomotic leak, Double-stapling technique, Staple-line intersection

## Abstract

**Aim:**

To systematically classify and describe transabdominal surgical techniques designed to eliminate or neutralize staple-line intersection during low anterior resection (LAR), and to summarize their reported clinical outcomes.

**Methods:**

A systematic review was conducted according to PRISMA guidelines and registered in PROSPERO (CRD420261368367). Major databases were searched for studies describing transabdominal techniques aimed at eliminating or neutralizing staple-line intersection in colorectal anastomosis during LAR. Both modified double-stapling techniques (mDST) and abdominal single-stapled techniques (aSST) were included. Outcomes of interest included anastomotic leak (AL), use of protective stoma, and perioperative variables. Reported outcomes were descriptively summarized, and exploratory aggregated analyses were performed when appropriate.

**Results:**

Seventeen studies (998 patients) were included. Ten studies (529 patients) evaluated mDST (suture-based: *n* = 192; invagination-based: *n* = 337), while seven studies (469 patients) assessed aSST. Reported AL rates remained low across both mDST subgroups (3.1% suture-based vs 3.9% invagination-based), with an aggregated rate of 3.6%. The aSST group showed a leak rate of 6.0%. Protective stomas were used in 6.2% of mDST and 33.1% of aSST. Neoadjuvant therapy was used in 20.7% of aSST and 11.9% of mDST. Considerable heterogeneity in study design, technique, and outcome reporting was observed.

**Conclusions:**

Transabdominal techniques that eliminate or neutralize staple-line intersection appear feasible and associated with low anastomotic leak rates. Modified DST techniques appear technically feasible and have been associated with low reported leak rates. However, owing to the heterogeneity of the available evidence, comparative findings should be considered exploratory.

## Introduction

Anastomotic leakage (AL) in rectal surgery remains one of the most feared complications, with reported incidence rates ranging from approximately 3% to over 20%, depending on patient, disease, and procedure-related factors [1, 2]. From a technical perspective, the introduction of stapling devices has revolutionized colorectal surgery by enabling safe and reproducible low pelvic anastomoses. The double-stapling technique (DST), originally described by Knight and Griffen [3], has become the most widely adopted approach following low anterior resection (LAR), as it overcomes the technical challenges associated with placing a distal purse-string suture in a narrow pelvis. However, DST inherently results in the intersection between the linear staple line used to close the rectal stump and the circular staple line used to construct the anastomosis. This configuration generates two lateral protrusions at the rectal stump edges (“dog-ears”), along with a crossing staple-line zone that may represent a mechanically and biologically vulnerable segment. Specifically, intersecting staple lines can lead to local tissue overcompression, irregular apposition, and impaired microvascular perfusion, potentially predisposing the anastomosis to structural weakness and subsequent leakage [4].

In response to these limitations, various strategies have been proposed to reduce the risk of AL. On one hand, reinforcement techniques aim to strengthen colorectal anastomoses, particularly when pre- and intraoperative assessment suggests an increased risk of leakage [5, 6]. On the other hand, several technical modifications have been developed to minimize or eliminate staple-line intersection. These include modified DST (mDST) techniques that address intrinsic weak points by removing, centralizing, invaginating, or reorienting the distal staple line before circular firing. These approaches encompass mattress or traction sutures, dog-ear invagination techniques, selective excision of one or both dog-ears, and geometric modifications of rectal transection designed to minimize the burden of crossing staple lines [7–16]. A second, more radical strategy consists in completely avoiding staple-line intersection through single-stapled anastomosis (SST). In this approach, the distal rectum is managed with a purse-string suture rather than linear stapling, allowing the anastomosis to be constructed using a single circular stapler thereby eliminating intersecting staple lines. Variants of SST include double purse-string techniques, intracorporeal purse-string approaches [17, 18], and more recent robotic intracorporeal reconstructions [19, 20]. The common rationale underlying these techniques is the creation of a more homogeneous, end-to-end anastomotic ring, free of crossing staples and residual lateral weak points, with the aim of improving structural integrity and perfusion [17, 18, 21].

The available literature is highly heterogeneous, with varied techniques, terminology, indications, and outcome reporting, limiting comparability and definitive conclusions. This systematic review aims to synthesize current evidence on surgical techniques by transabdominal approach to eliminate or neutralize staple-line intersection in colorectal anastomosis in the context of LAR, to provide a technical classification of these procedures and summarize the currently available clinical evidence.

## Methods

### Study design and search strategy

An extensive bibliographic search of pertinent English literature was performed according to PRISMA criteria. The protocol of this study was prospectively registered in the International Prospective Register of Systematic Reviews (PROSPERO) (registration code CRD420261368367). Scopus, ScienceDirect, Web of Science, MEDLINE, PubMed, and the Cochrane Database were systematically searched to identify studies describing techniques aimed at eliminating or neutralizing staple-line intersection in colorectal anastomosis. Studies reporting surgical techniques aimed at eliminating or neutralizing staple-line intersection or modifying the standard DST configuration in colorectal anastomosis during LAR were included. All clinical, experimental, or technical studies providing a clear description of the surgical technique were considered eligible. Exclusion criteria comprised non-English articles, duplicates, studies not related to colorectal anastomosis during LAR, studies exclusively involving transanal approaches (e.g., TTSS or TaTME), and abstract-only publications or unavailable full texts.

For the purpose of this review, “eliminate or neutralize staple-line intersections” was defined as any surgical technique that prevents or eliminates the crossing between linear and circular staple lines during rectal anastomoses. This includes both modified DST and SST approaches. mDST include procedures involving at least two stapler firings—a linear stapler for rectal transection and a circular stapler for anastomosis—in which specific technical modifications are applied to eliminate or neutralize the formation of dog-ears between rectal transection and circular stapling. These techniques can be further subclassified according to the underlying mechanism into *suture-based techniques*, in which stitches are used to resect, approximate, or neutralize the dog-ears before construction of the anastomosis, or *invagination techniques*, in which the lateral extremities of the rectal stump are inverted or incorporated into the anastomotic lumen prior to circular stapling. Techniques were classified according to the actual anastomotic configuration rather than the terminology used by the original authors. In example, despite being described by the authors as a “single stapled” configuration, the Bow Tie technique still involves rectal linear stapling and was therefore categorized as mDST for the purpose of this review [16]. Conversely, abdominal SST (aSST) refers to all techniques in which a purse-string suture is performed, and a single circular stapler firing is used to construct the colorectal anastomosis. Within this category, the abdominal approach specifically indicates that both rectal transection and purse-string construction are carried out via a transabdominal route.

The methodological quality of the included studies was evaluated using the modified Newcastle–Ottawa Scale (NOS) for cohort studies [22]. The NOS assigns up to sixteen points across four domains: Clearness of the Aim (two points), Sample Selection (eight points for representativeness, sample size, non-response rate and exposure assessment), Comparability (two points for Control of confounding factors and Comparability of groups), and Outcome (four points for assessment of the outcome and statistics). Each item was judged as either fulfilled (awarding a star) or not fulfilled. Two authors (AA and GGa) independently assessed all studies, with disagreements resolved by discussion with the third author (AP). Based on the total score, studies were categorized as high quality and low risk of bias (13–16 points), moderate risk of bias (9–12 points) and low quality and high risk of bias (< 9 points).

The primary aim of this study was to evaluate the AL rates achieved with transabdominal techniques designed to eliminate or neutralize staple-line intersection during colorectal anastomosis after low anterior resection. Additional outcomes, including the use of protective ostomy and perioperative variables, were also considered. Where sufficient data were available, descriptive aggregated leak rates were performed by aggregating event counts (AL, ostomy rates) across predefined subgroups (suture-based mDST, invagination-based mDST, and aSST). Studies with missing or non-extractable data for specific outcomes were excluded from the corresponding aggregated analyses. Because of the expected methodological and clinical heterogeneity among studies—including differences in study design, patient selection, neoadjuvant treatment, diverting stoma use, and reporting of anastomotic height—a formal meta-analysis was considered inappropriate. Therefore, only descriptive aggregated analyses of crude event rates were performed to summarize the available evidence.

### Descriptive aggregated analysis

A descriptive aggregated analysis was performed to estimate event rates across studies. Given the marked heterogeneity and predominance of non-comparative studies, only descriptive aggregated analyses of crude event rates were performed without weighted meta-analytic modelling. For each outcome, the number of events and the total number of patients were summed within each predefined subgroup (suture-based mDST, invagination-based mDST, and aSST), and proportions were calculated accordingly. For variables with incomplete reporting (i.e., protective ostomy and neoadjuvant therapy), only studies providing the relevant data were included in the denominator for that specific analysis. Aggregated rates were reported with corresponding 95% confidence intervals (95% CI).

## Results

**Fig. 1:**
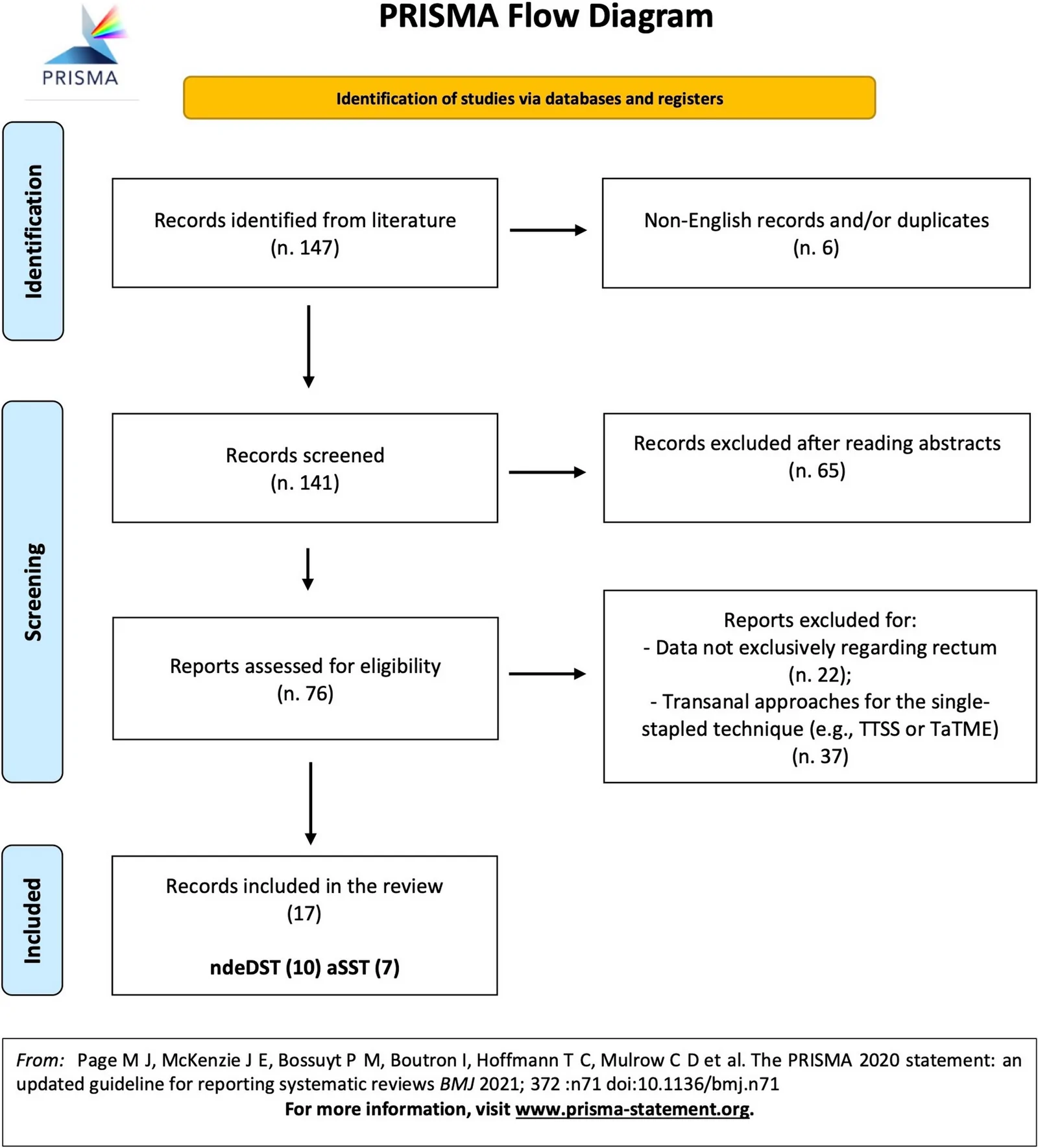
PRISMA figure of included studies

A total of 147 records were identified from the literature, 17 studies were finally included in the qualitative synthesis (998 patients; Table 1, Fig. 1) [7–21, 23, 24]. Study designs were heterogeneous and involved technical notes, retrospective series, prospective studies and randomized controlled trials (Table 1). Definitions of AL were inconsistently reported, with several technical reports not providing standardized outcome definitions (Table 1). There were 10 studies on mDST techniques (529 patients) [7–16], and 7 studies on aSST techniques (469 patients) (Table 1) [17–21, 23, 24]. mDST were further subclassified into suture-based techniques (192 patients) [7, 9, 13, 14] and invagination-based techniques (337 patients; Table 1) [8, 10–12, 15, 16].

**Table 1:** Characteristics of studies included in the review. *T*, technical note; *R*, retrospective; *P*, prospective; *RCT*, randomized controlled trial; *NR*, not reported; *mDST*, non-dog-ears Double Staple Technique; *aSST*, abdominal Single Stapled Technique; *DAIA*, Dog Ear Invagination Anastomosis; *MDST*, Modified Double Stapled Technique; *nDST*, new Double Stapled Technique; *No*, number; *NR*, not reported; * From Anal Verge; °From Dentate Line. *Lap.*, laparoscopic; *Robot.*, robotic. Continuous parameters are reported as mean (standard deviation) or median (range), as originally reported. Distances were extracted as reported in the original studies and may refer to the anal verge or dentate line

Category	Authors	Year	Technique (subtype)	Type of study	Time recruitment	Definition of anastomotic leak	No.of patients	Neoadjuvant therapy (%)	Gender (males)	Distance/height as reported (cm)	Approach	Operative time min. (std)	Anastomotic leaks *n* (%)	Protective ostomy	Length of stay (days)
mDST suture	Asao et al. [7]	2002	Omega Mattress Suture	T	NR	NR	6	NR	5 (83.3%)	NR	Lap. = 3; Open = 3	NR	0 (0)	NR	NR
	Chen et al. [9]	2016	Dog-Ear’s Suturing	R	2012–2014	NR	48	NR	NR	NR	Lap	130.5 (100–180)	1 (2.1)	0 (0)	NR
	Yang et al. [13]	2022	MDST	RCT	2015–2018	Reported	116	0	70 (60.3%)	11.6 ± 3.2 *	Lap	126 (114–145)	4 (3.4)	0 (0)	6.7 ± 1.6
	Kim et al. [14]	2024	nDST	R	2018–2020	Reported	22	0	13 (59.1%)	9.5 ± 2.4 *	Lap	123 ± 28.5	1 (4.6)	0 (0)	6.3 ± 1.0
mDST invagination	Zhuo et al. [8]	2015	Rectal Eversion	T	2014–2015	NR	11	4	6 (54.5%)	4.7 ± 1.3°	Lap	191 ± 44.7	0 (0)	8 (72.7)	8.8 ± 2.3
	Crafa et al. [10]	2017	TICRANT Technique	P	2013–2016	Reported	53	22	35 (66.0%)	8 (4–12) *	Open = 30; Lap. = 16; Robot. = 7	NR	3 (5.7)	NR	NR
	Zhang et al. [11]	2018	DAIA	R	2015–2016	NR	18	4	8 (44.4%)	5 (3–7.5) °	Lap	150 (120–180)	0 (0)	0 (0)	NR
	Zhang et al. [12]	2022	DAIA	R	2015–2017	Reported	53	NR	34 (64.2%)	8.6 ± 2.7 *	Lap	148.8 ± 10.3	2 (3.8)	3 (5.7)	8.9 ± 2.8
	Liu et al. [15]	2025	Dog-Ear Inclusion	P	2022–2023	Reported	140	0	NR	(5–16) *	Lap	NR	5 (3.6)	0 (0)	NR
	Van Zande et al. [16]	2025	Bow Tie Technique	R	2023–2024	Reported	62	20	37 (59.7%)	NR	Lap. = 60; Open = 2	128 (65–204)	3 (4.8)	18 (29.0)	6
aSST	Sato et al. [17]	2006	SST	R	1991–1999	NR	47	0	29 (61.7%)	7.1 (3–12) *	Open	245 (100–438)	6 (12.8)	19 (40.4)	NR
	Marecik et al. [21]	2007	SST	P	2000–2005	Reported	160	NR	64 (40.0%)	NR	Lap. = 45; Open = 115	NR	1 (0.6)	0 (0)	NR
	Shrikhande et al. [18]	2007	SST	R	1999–2005	Reported	78	0	56 (71.8%)	8 (4–5) *	Open	NR	8 (10.3)	51 (65.4)	NR
	Prasad et al. [19]	2010	SST	T	NR	NR	3	0	2 (66.7%)	NR	Robot	339.6	0 (0)	0 (0)	4.3 (4–5)
	Kim et al. [23]	2013	SST	R	2007–2010	Reported	60	5	26 (43.3%)	7.8 (3–12) *	Lap. = 23; Robot. = 37	203.9 (120–400)	6 (10)	0 (0)	NR
	Radovanic et al. [24]	2014	CSA	P	2007–2009	Reported	50	21	31 (62.0%)	8.8 (4–14) *	Open	141	3 (6)	14 (28)	NR
	Stevenato Filho et al. [20]	2025	SST	R	2018–2025	Reported	71	38	51 (71.8%)	5.1 ± 2.3°	Robot	327 ± 59	4 (5.6)	71 (100)	6 (3–21)

The overall methodological quality of the included studies was heterogeneous (Table 2). A substantial proportion of studies achieved high NOS scores [12–16, 18, 20, 23, 24], particularly recent comparative and prospective analyses, while several earlier reports were limited by small sample size and retrospective design. Moderate-quality studies lacked adequate adjustment for confounders or complete outcome reporting [9, 10, 17, 21]. Low-quality studies were mainly technical case series with limited external validity and incomplete methodological description [7, 11].

**Table 2:** Newcastle–Ottawa scale of included studies. *mDST*, non-dog-ears Double Staple Technique; *aSST*, abdominal Single Stapled Technique

Study		Clearness of stated aim (0–2)	Sample selection				Comparability		Outcome		Total	Quality
			Sample representativeness (0–2)	Sample size (0–2)	Non-respondents (0–2)	Exposure assessment (0–2)	Control of confounding factors (0–1)	Comparability of participants (0–1)	Assessment of the outcome (0–2)	Statistical tests (0–2)		
mDST suture	Asao et al. [7]	2	0	0	0	2	0	0	1	0	5	Low
	Chen et al. [9]	2	1	1	0	2	0	0	2	1	9	Moderate
	Yang et al. [13]	2	2	2	2	2	1	1	2	2	16	High
	Kim et al. [14]	2	2	1	1	2	1	1	2	2	14	High
mDST invagination	Zhuo et al. [8]	2	0	0	0	2	0	0	1	0	5	Low
	Crafa et al. [10]	2	2	1	1	2	0	0	2	1	11	Moderate
	Zhang et al. [11]	2	0	0	0	2	0	0	1	0	5	Low
	Zhang et al. [12]	2	2	2	1	2	1	1	2	2	15	High
	Liu et al. [15]	2	2	2	1	2	1	1	2	2	15	High
	Van Zande et al. [16]	2	2	2	1	2	0	0	2	2	13	High
aSST	Sato et al. [17]	2	1	1	0	2	0	0	2	1	9	Moderate
	Marecik et al. [21]	2	1	2	0	2	0	0	2	1	10	Moderate
	Shrikhande et al. [18]	2	2	2	1	2	1	1	2	2	15	High
	Prasad et al. [19]	2	0	0	0	2	0	0	1	0	5	Low
	Kim et al. [23]	2	2	1	1	2	1	1	2	2	14	High
	Radovanic et al. [24]	2	2	2	2	2	1	1	2	2	16	High
	Stevenato Filho et al. [20]	2	2	2	1	2	1	1	2	2	15	High

### Suture-based mDST

**Fig. 2:**
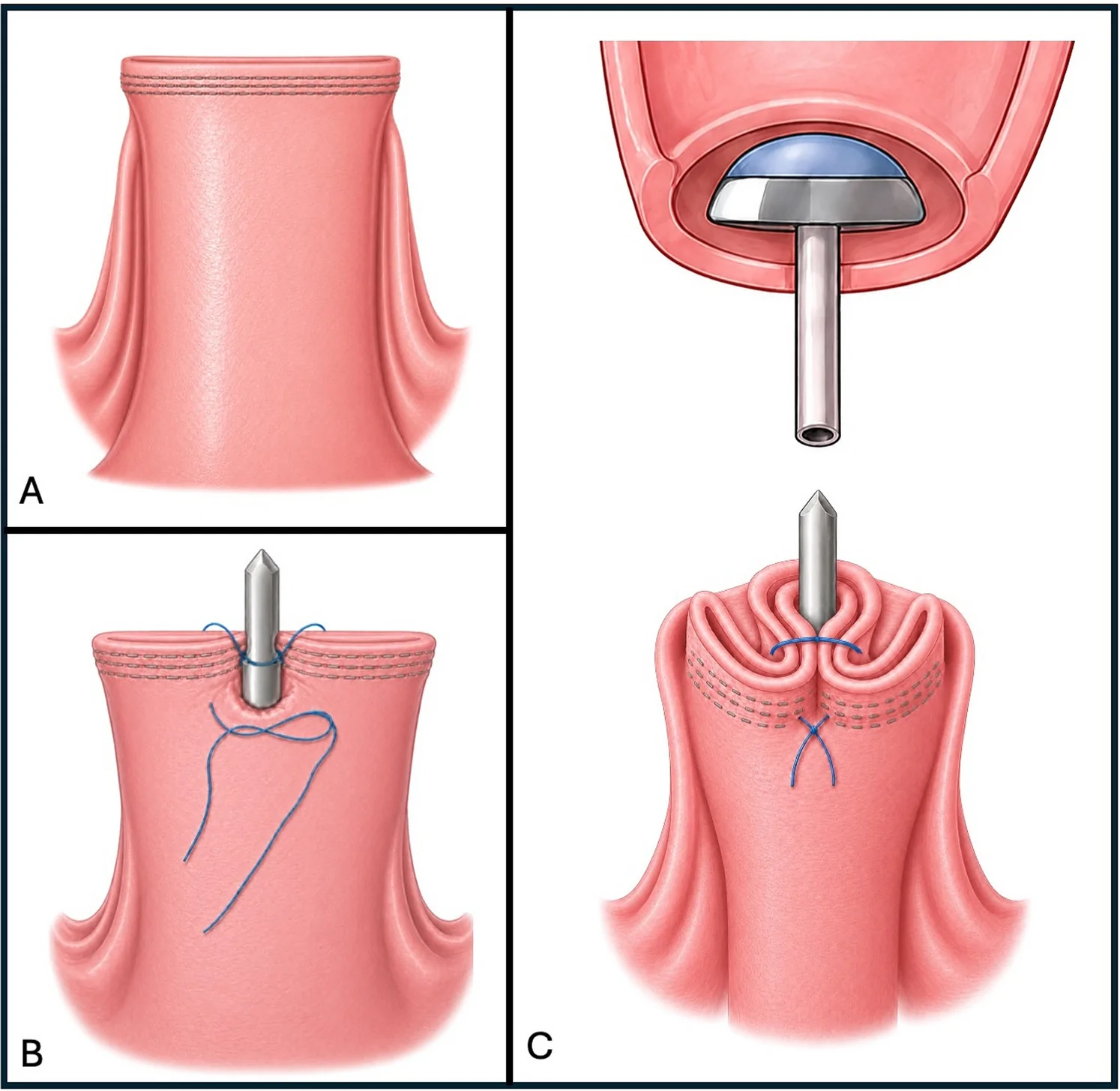
Omega mattress suture technique. After rectal transection (**A**), a running mattress suture was placed across the central portion of the staple line. The suture was tied around a temporary dummy shaft (**B**), converting the straight staple line into an omega-shaped configuration that approximated both lateral dog-ears. The remodeled staple line was then completely encompassed by the circular stapler, allowing excision of the entire linear staple line during firing (**C**)

**Fig. 3:**
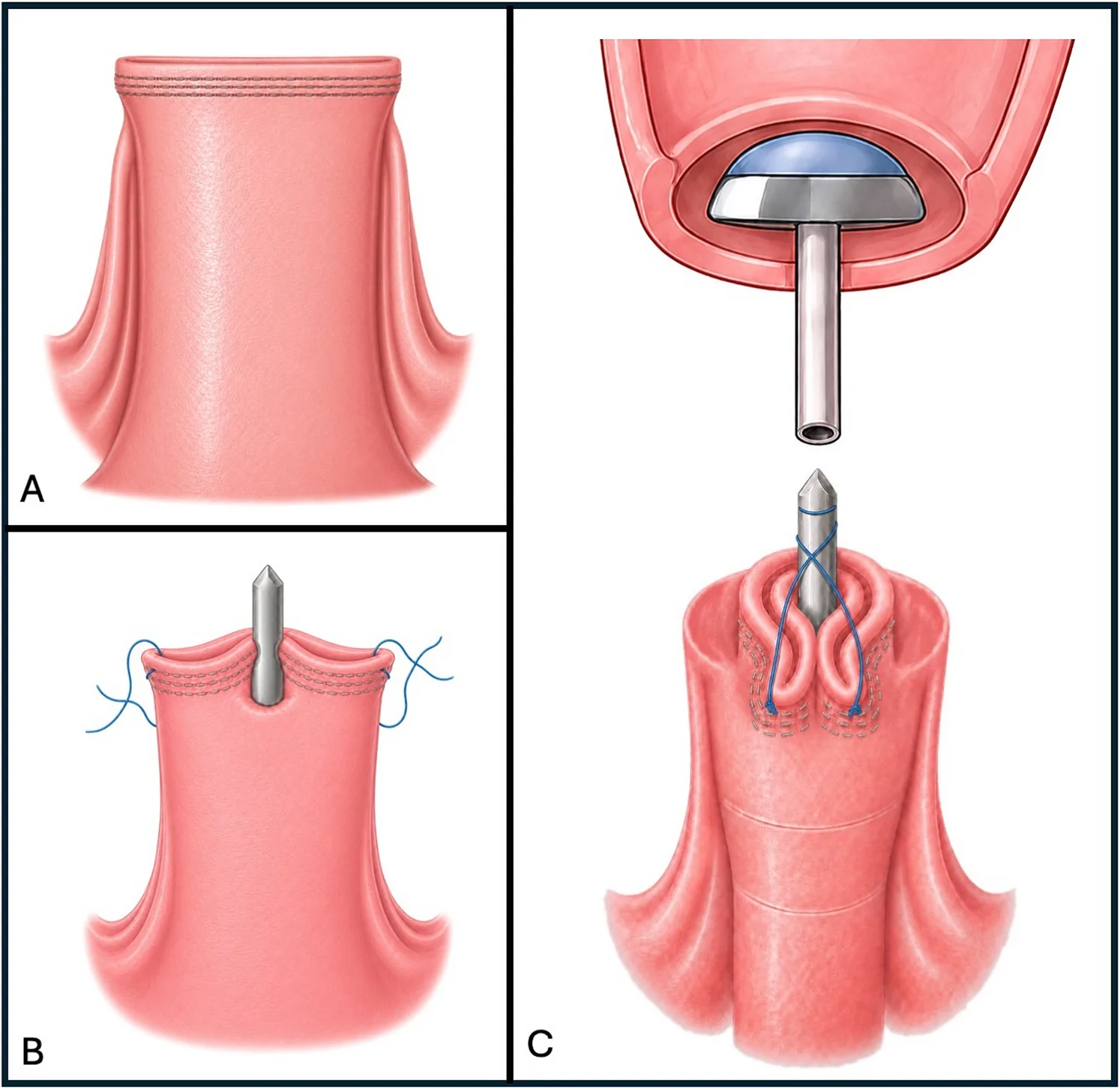
Dog-ear suturing technique. Following standard DST rectal transection (**A**), the two lateral dog-ears were identified at the extremities of the staple line. Laparoscopic sutures (1–0 or 2–0 monofilament) were placed on each dog-ear (**B**) and temporarily secured around the central shaft of the circular stapler, pulling them toward the center of the rectal lumen (**C**). This allowed incorporation and resection of the dog-ears during anastomosis

**Fig. 4:**
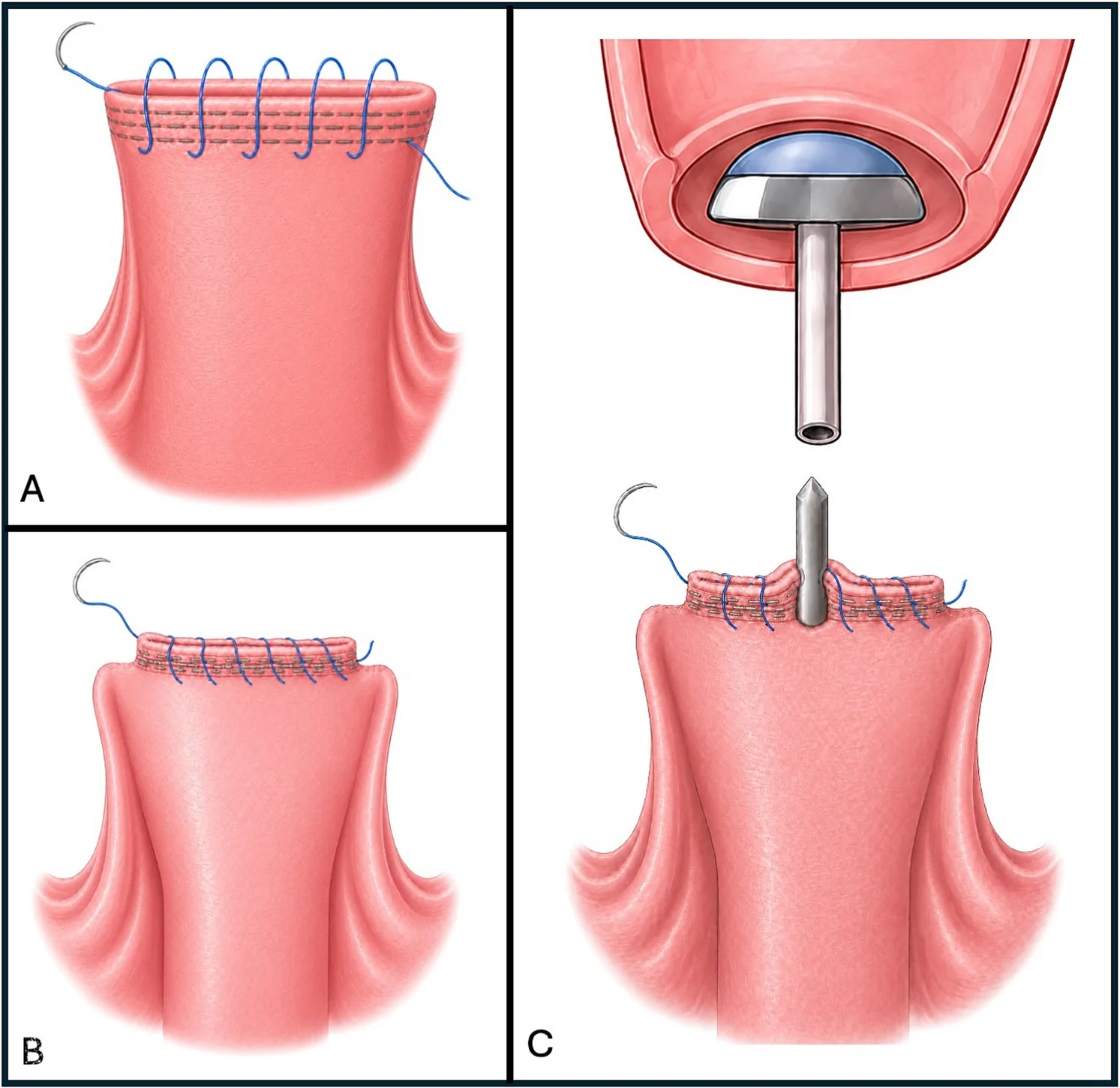
New double-stapling technique. Rather than directly manipulating the dog-ears, this technique focuses on preventing their formation. After rectal transection, the linear staple line was shortened using a continuous barbed suture (V-Loc 3-0) (**A**) which is pulled shortening the staple line (**B**). This reduced its length and eliminated lateral protrusions, preventing the formation of dog-ears and avoiding staple-line intersection during circular stapling (**C**)

*Omega mattress suture technique:* After linear transection, a central running suture tied around a midpoint dummy shaft reshapes the staple line into an “omega,” collapsing the lateral edges. The remodeled line is then fully captured by the circular stapler allowing complete incorporation of the entire linear staple line within the circular stapler (Fig. 2) [7]. *Dog-ear suturing technique:* the dog-ears are sutured and drawn toward the central shaft of the circular stapler. This maneuver centralizes them within the lumen, allowing their incorporation and resection during anastomosis (Fig. 3) [9, 13]. *New double-stapling technique:* After rectal transection, a continuous barbed suture progressively shortened the linear staple line, converting it into a shorter curved configuration. This maneuver eliminated the lateral dog-ears before circular stapling, thereby preventing staple-line intersection (Fig. 4) [14].

All procedures were performed laparoscopically except for 3 open procedures in the earliest study [7]. The overall mean operative time was 126.8 min (Table 1). The incidence of AL ranged from 0% to 4.6%, with low variability across studies [7, 14]. Protective stomas were generally not used in these series, exception made for Asao et al. [7].

### Invagination-based mDST

**Fig. 5:**
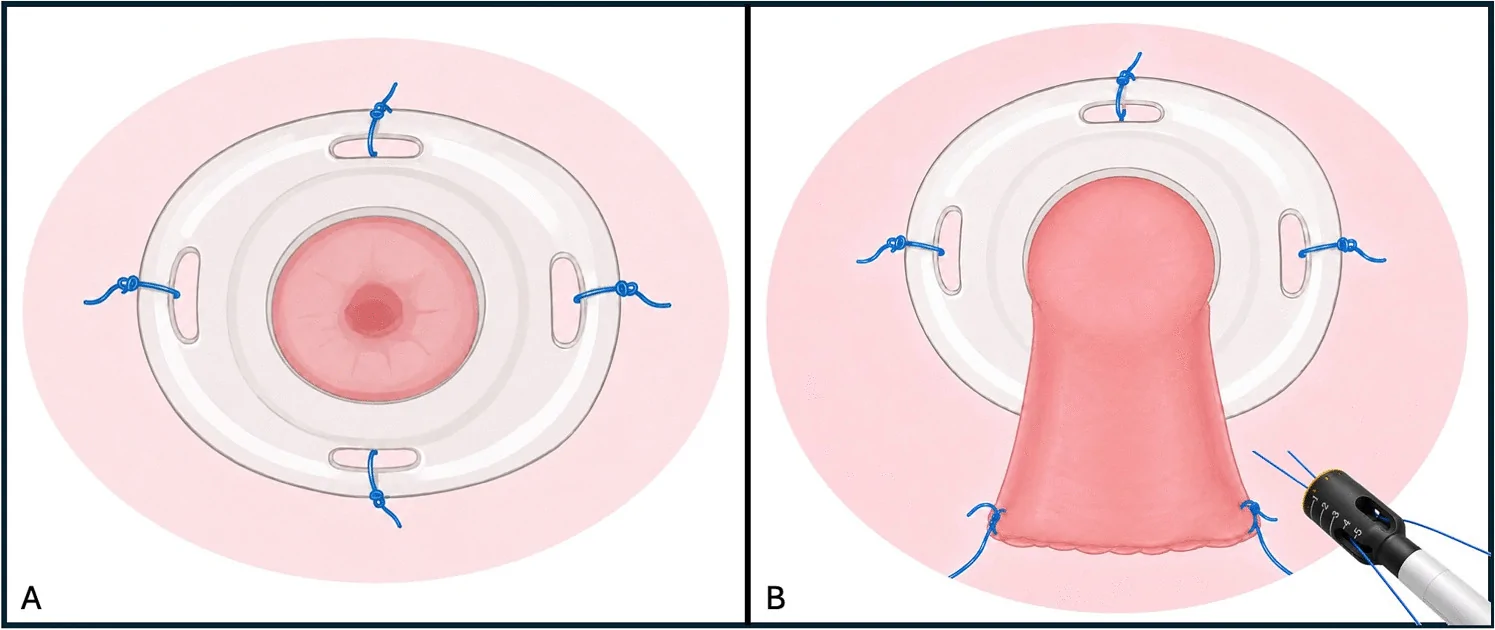
Rectal eversion and resection technique. After laparoscopic rectal transection (**A**), the distal rectum was everted transanally allowing direct visualization of the staple line (**B**). A second linear stapling was performed under direct vision to ensure an adequate distal margin. Two 2-0 Prolene stay sutures were placed at both ends of the resection line and their tails were passed through the lateral windows of the stapler (**B**) [8]. Progressive traction was applied to these sutures resulting in invagination of the dog-ears toward the center of the lumen. The rectal stump including the dog ears was completely loaded into the cartridge of the stapler

**Fig. 6:**
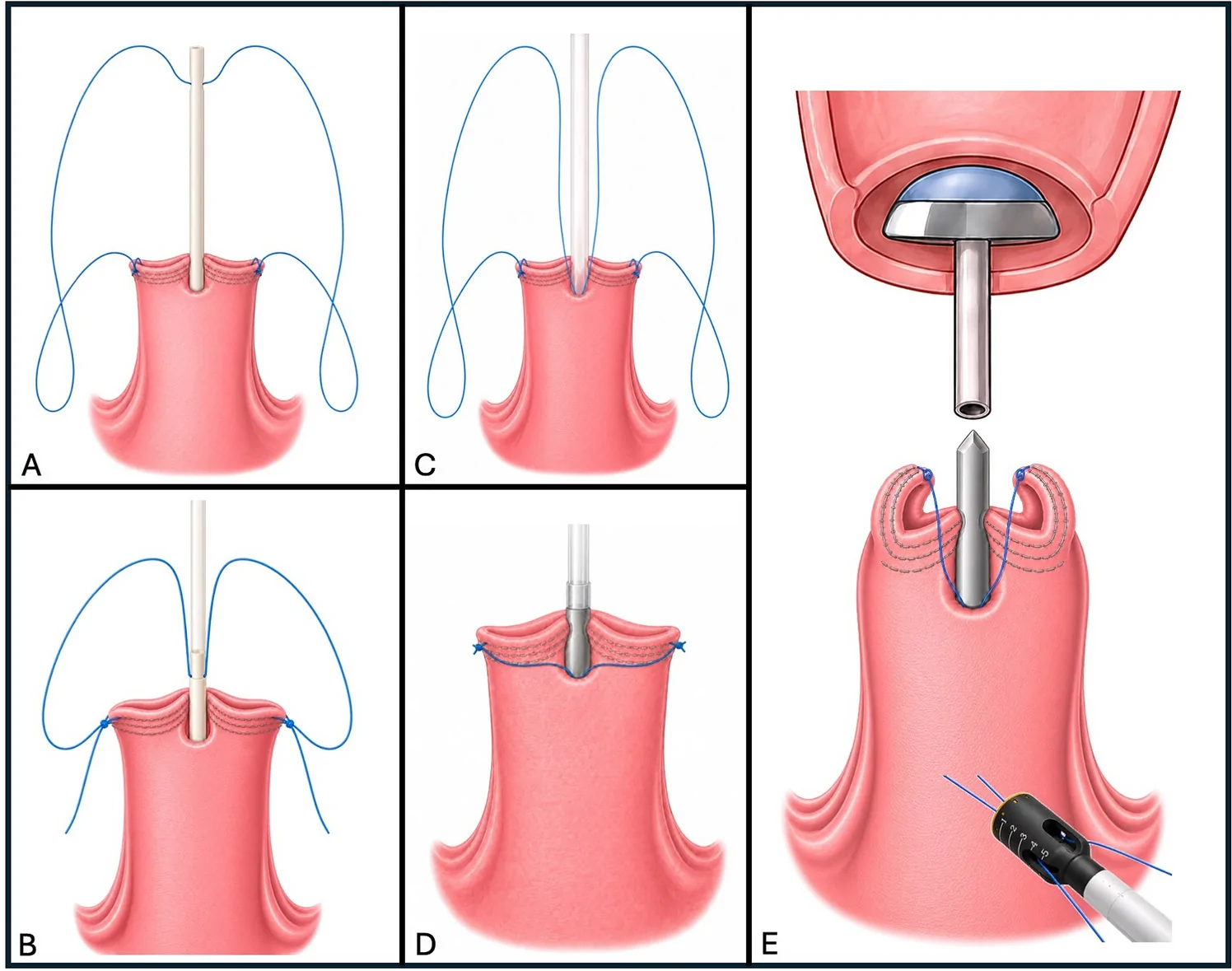
Dog-ear invagination anastomosis (DAIA). After standard DST, two 2-0 Prolene sutures were placed on the two stapled extremities. A special trocar was introduced transanally through the middle of the rectal stump, and the sutures were connected to the pre-configured notches in the special trocar (**A**). A surgical drainage tube was introduced into the abdomen and connected to the special trocar which was withdrawn, pulling the surgical drainage tube and the sutures through the anus (**B**, **C**). Then the catheter was detached, the sutures which were introduced in the windows of the stapler and the anvil was reintroduced in the abdomen with the help of the drainage (**D**). The sutures previously inserted through the stapler traction holes were gently pulled during the closure of the stapler to obtain a gradual and homogeneous traction of the tissue and elimination of both suture line and dog-ears (**E**)

**Fig. 7:**
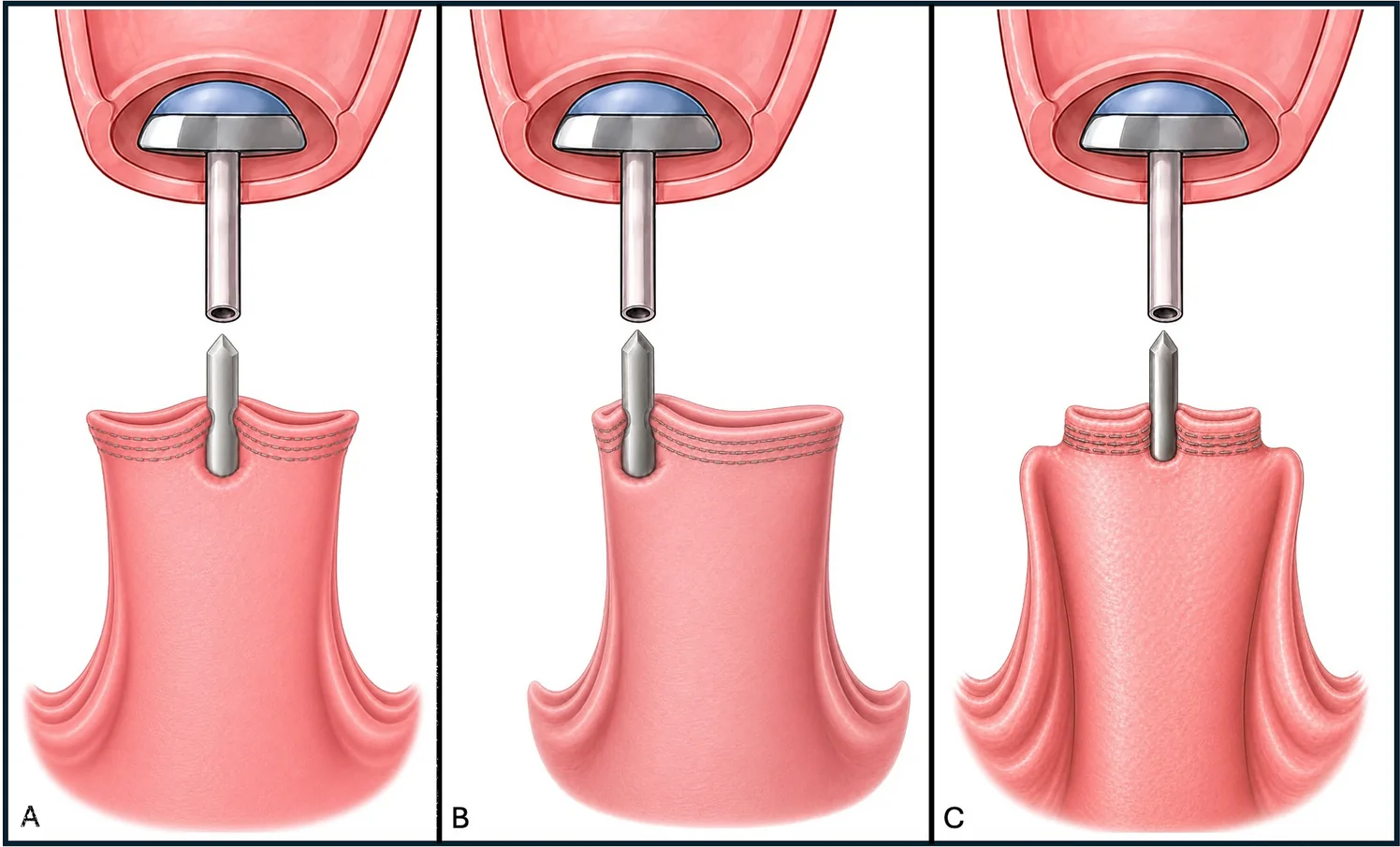
Selective dog-ear resection technique. In normal situation both dog-ears were excluded by the firing (**A**). In this approach the angle and direction of the stapler’s central shaft were adjusted to ensure that the anvil ring would encompass at least one of the dog-ear areas (**B**). When the conformation of the linear suture line allows it, efforts were made to include both dog-ears within the stapling ring, enabling complete excision of the dog-ears (**C**)

**Fig. 8:**
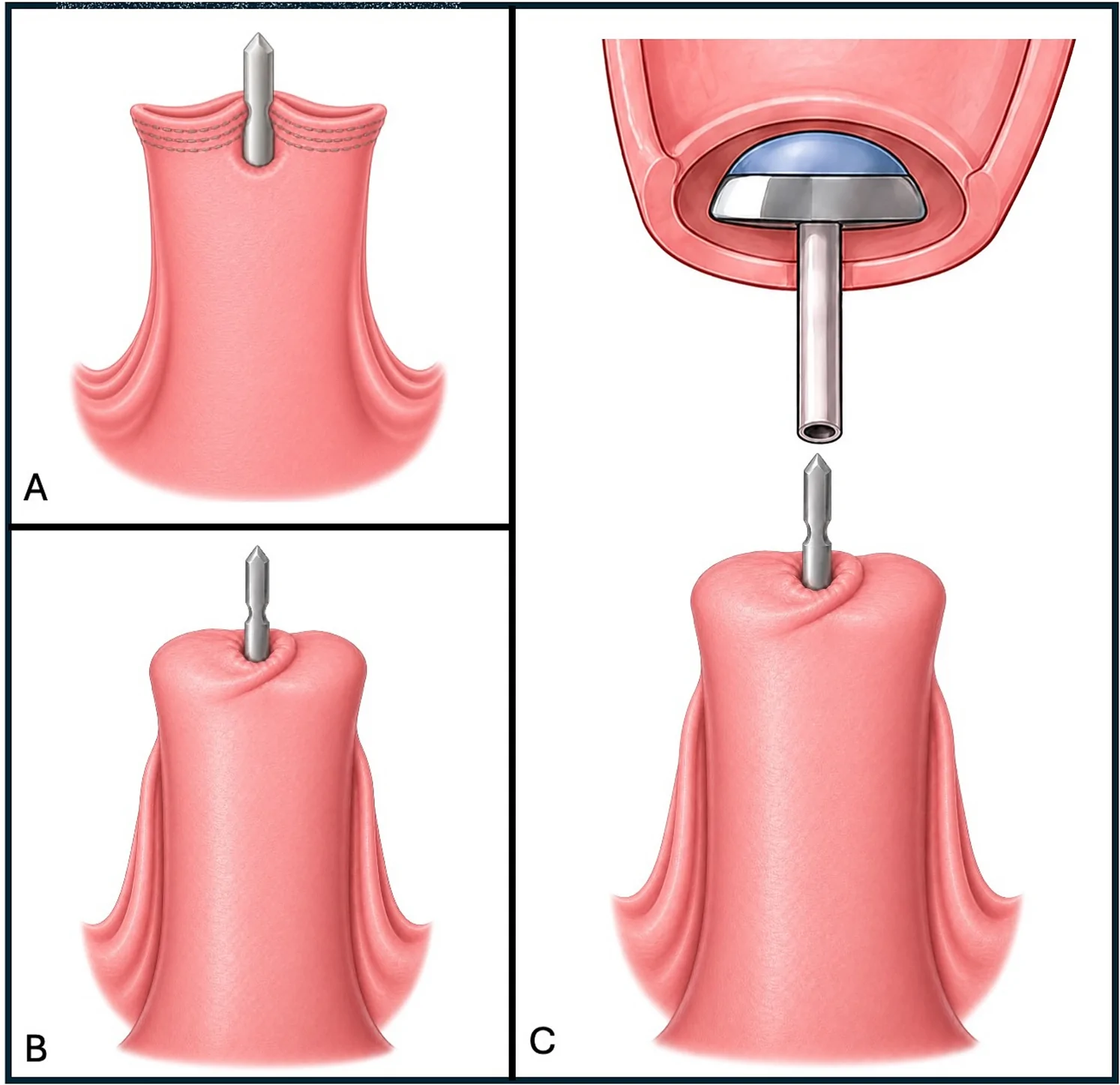
Bow-Tie technique. After standard linear resection, the stapler spike was introduced centrally through the staple line, (**A**) and both dog-ears were manually invaginated and fixed onto the spike (**B**). This created a “bow tie” configuration in which the linear staple line was entirely encompassed by the circular stapler (**C**). Firing resulted in complete resection of the dog-ears

*Rectal eversion and resection technique:* After laparoscopic transection, the rectum is everted transanally and a second linear stapling is performed under direct vision. Stay sutures at both ends are passed through the stapler, allowing complete incorporation of the stump, including dog-ears, into the cartridge (Fig. 5) [8]. *TICRANT technique:* unlike the Zhuo technique, no second rectal transection is performed. Following transanal eversion, the original rectal staple line is inspected under direct vision and traction sutures are placed at its extremities, allowing invagination of the existing staple line into the circular stapler [10]. *Dog-ear invagination anastomosis (DAIA):* two traction sutures were placed on both dog-ears. A dedicated transanal trocar was then used to exteriorize these sutures through the anal canal, allowing symmetrical traction of both dog-ears while closing the circular stapler. This progressively invaginated the lateral staple-line extremities into the stapler cartridge (Fig. 6) [11, 12]. *Selective dog-ear resection technique:* Instead of centering the circular stapler on the rectal lumen, the stapler shaft was intentionally directed toward one or both dog-ears so that they were selectively included within the stapling ring and excised during firing (Fig. 7) [15]. *Bow-Tie technique:* Both dog-ears were invaginated and fixed onto the shaft spike before firing creating a symmetric bow-tie configuration before stapling (Fig. 8) [16].

Most procedures were performed laparoscopically with some studies including open and robotic approaches [10, 16]. The reported anastomotic leak rate ranged from 0% to 5.7% [8, 16], with variability likely related to differences in patient selection and study design. The use of protective ostomy varied considerably across studies, ranging from 0% to 72.7% [11, 15].

### aSST techniques

**Fig. 9:**
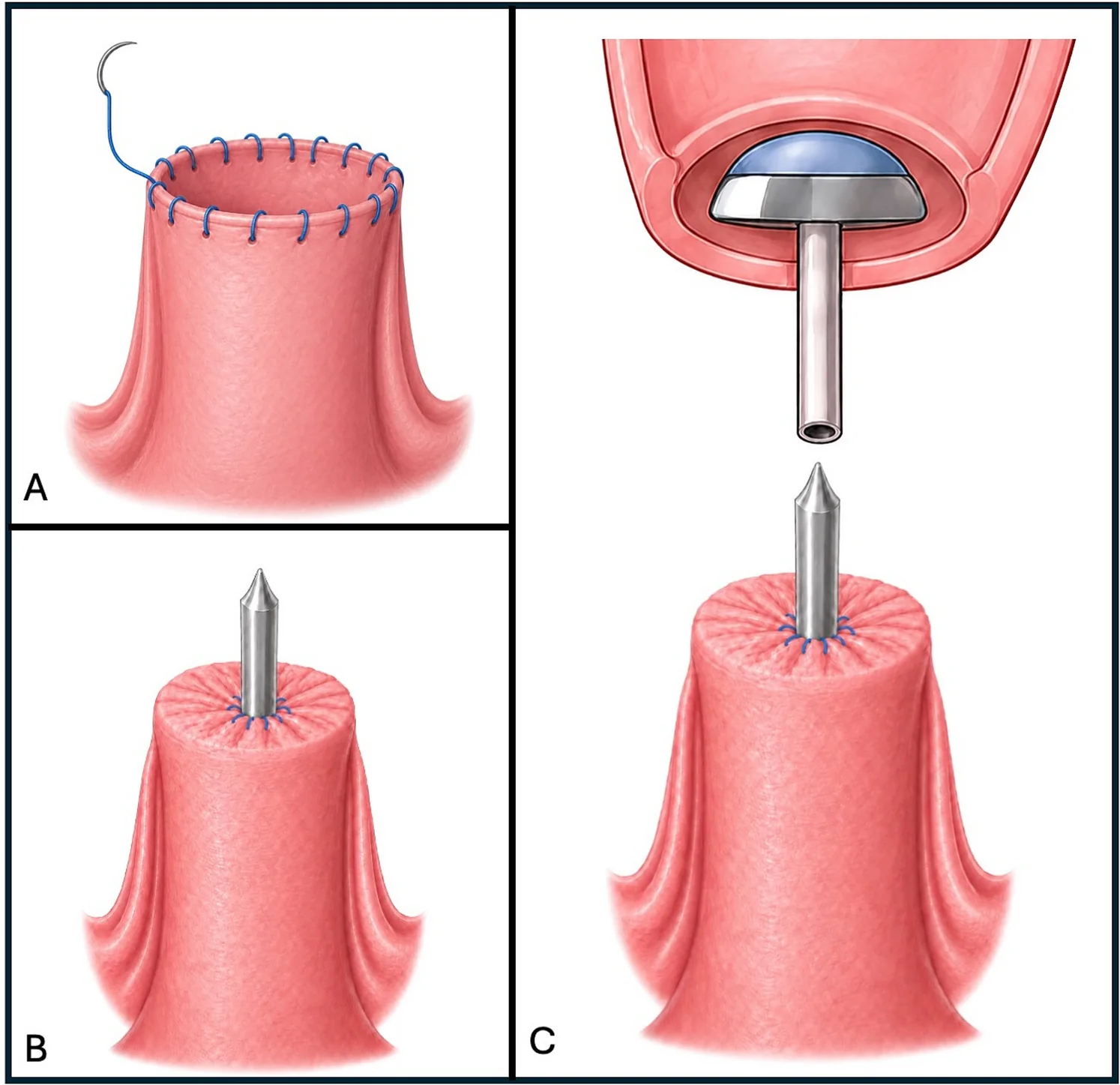
Single-Stapled colorectal anastomosis. After distal rectal transection, the rectal stump is prepared with a circumferential purse-string suture, which may be placed manually, laparoscopically, robotically, transanally, or with a dedicated purse-string device. Rectal washout and distal control with a clamp may be performed before transection (**A**). The purse-string suture is tightened around the central shaft of the circular stapler introduced transanally, allowing the rectal stump to be closed around the stapler spike under direct vision (**B**). After engagement with the proximal anvil, the circular stapler is closed and fired, completing a single-stapled colorectal anastomosis (**C**). This sequence summarizes the key steps reported across different technical variations, including hand-sewn, automated, laparoscopic, robotic, and transanal purse-string approaches. The exact sequence may vary according to the specific technique

*aSST:* Although several technical variants have been described, all aSST techniques share the same principle: the distal rectum is closed with a purse-string suture instead of a linear stapler, thereby eliminating the linear staple line and any staple-line intersection before circular stapling. Variations mainly concern how the purse-string is created (manual, laparoscopic, robotic, transanal or using dedicated devices). More specifically, the manual purse-string suture is placed on the distal stump following division of the rectum, then the circular stapler introduced and the purse-string tied around the central shaft either directly (Fig. 9) [17, 18], or using a “dummy-head” protector [21]. Purse string sutures can also be performed using a reusable purse-string instrument [24]. As an alternative, the colon and rectum are ligated at the planned resection margins, the rectum is washed with Betadine, and both segments are divided with electrocautery at least 1 cm from the ligatures. Colon and rectal purse-strings are then performed, the specimen extracted transanally, the anvil inserted, and the anastomosis completed [14]. Sutures can also be performed robotically after a transanal purse-string suture is placed to isolate the distal stump, before rectal transection with monopolar hook or robotic scissors [19, 20]. A robotic intracorporeal purse-string is placed and a circular stapler introduced to complete the anastomosis [19, 20].

Both open and minimally invasive approaches were reported, with increasing adoption of laparoscopic and robotic techniques in more recent studies [14, 19, 20]. The reported anastomotic leak rate ranged from 0% to 12.8% [17, 19], with higher variability compared to DST techniques. The use of protective stomas was highly heterogeneous, ranging from 0 to 100% [14, 19–21], particularly in more recent robotic series [20].

#### Descriptive aggregated analysis

Among studies reporting neoadjuvant therapy data, no patients received neoadjuvant treatment in the suture-based mDST subgroup (0/138, 0%; 95% CI 0.0–2.7) [13, 14], while in the invagination-based subgroup the rate was 17.6% (50/284; 95% CI 13.6–22.5) [8, 10, 11, 15, 16]. When both mDST subgroups were combined, the aggregated neoadjuvant therapy rate was 11.9% (50/422; 95% CI 9.0–15.4) [8, 10, 11, 13–16]. The aSST group showed high rates of neoadjuvant therapy, reaching 20.7% (64/309; 95% CI 16.5–25.7) [14, 17–20, 24].

Among studies reporting protective ostomies, none were observed in the suture-based mDST subgroup (0/186, 0%; 95% CI 0.0–2.0) [9, 13, 14], while in the invagination-based subgroup the ostomy rate was 10.2% (29/284; 95% CI 7.2–14.3) [8, 11, 12, 15, 16]. When both mDST subgroups were combined, the aggregated ostomy rate was 6.2% (29/470; 95% CI 4.3–8.7) [8, 9, 11–16]. In contrast, the aSST group showed a ostomy rate of 33.1% (155/469; 95% CI 28.9–37.4) [17–21, 23, 24].

The anastomotic leak rate was 3.1% (6/192; 95% CI 1.5–6.7) in the suture-based DST group [7, 9, 13, 14], and 3.9% (13/337; 95% CI 2.3–6.5) in the invagination-based group [8, 10–12, 15, 16], showing similar reported leak rates (Table 2). When both DST techniques were considered together, the overall leak rate was 3.6% (19/529; 95% CI 2.3–5.5) [7–16]. In contrast, the SST group showed a leak rate of 6.0% (28/469; 95% CI 4.2–8.5) (Table 2) [17–21, 23, 24].

## Discussion

Stapling devices have revolutionized colorectal surgery by enabling safe and reproducible pelvic anastomoses. The double-stapling technique, originally described by Knight and Griffen [3], is currently the most widely adopted approach, but its intrinsic configuration results in the intersection between linear and circular staple lines and generates potentially vulnerable ischemic zones (lateral “dog-ears”). Some recent transanal series have suggested lower leak rates with SST configurations compared with conventional DST [25]. The rationale of the present review was not to compare mDST with aSST, but rather to provide a mechanism-based overview of all transabdominal strategies designed to eliminate or neutralize staple-line intersection and to summarize the clinical outcomes achieved with each approach. Within this framework, mDST and aSST represent two conceptually different reconstructive strategies that pursue the same technical endpoint, namely elimination or neutralization of staple-line intersection. This distinction is clinically relevant because the transabdominal approach is familiar, standardized, and widely adopted in contemporary rectal surgery.

In our aggregated analysis, the leak rate was 3.1% (6/192) in suture-based mDST and 3.9% (13/337) in invagination-based mDST, suggesting similar reported leak rates between the two approaches. When both modified DST subgroups were considered together, the overall leak rate was 3.6% (19/529); in contrast, the aSST group showed a leak rate of 6.0% (28/469). Owing to the substantial heterogeneity of the included studies and baseline imbalances, no conclusions regarding comparative effectiveness can be drawn. Nevertheless, if confirmed in adequately designed comparative studies, these observations are of interest because SST theoretically provides a more homogeneous anastomotic configuration, with no linear rectal staple line, no staple-line intersection, and no residual dog-ears. The technical complexity of distal purse-string construction, however, may offset these theoretical advantages. At the same time, mDST techniques retain the practical benefits of conventional DST while addressing its principal structural weakness. This interpretation is consistent with recent reports describing favorable outcomes with mDST approaches [11, 13–15].

The similar leak rates observed between suture-based and invagination-based mDST suggest that the specific method may be less important than the achievement of the same technical endpoint: complete elimination or neutralization of the lateral staple-line extremities. This is consistent with recent evidence showing that anastomotic configuration, staple-line crossing, and the number of stapler firings may influence leakage risk after rectal surgery [26, 27]. Still, these findings must be interpreted cautiously. The comparison is affected by relevant confounders, including types of studies, diverting stoma use, tumor height, neoadjuvant therapy, and baseline patient risk. In our dataset, protective stomas appeared in 6.2% of the mDST group and 33.1% of the aSST group, which may reflect selection of higher-risk patients toward lower tumors and technically more demanding anastomoses. Moreover, diversion may reduce the clinical consequences of leakage and reoperation rates, but its effect on the true biological occurrence of leakage remains complex and heterogeneous across studies [28, 29]. Similarly, neoadjuvant therapy was observed in 20.7% of the aSST group and 11.9% of the mDST group and represents a relevant source of baseline imbalance, as preoperative chemoradiotherapy is a well-established risk factor for impaired tissue healing and anastomotic leakage [30]; therefore, the comparison of leak rates between techniques should be interpreted with caution.

### Strength and limitations

To our knowledge, this is the first systematic review proposing a mechanism-based classification of transabdominal techniques specifically designed to eliminate or neutralize staple-line intersection. Furthermore, a methodological strength of this review is that we restricted inclusion to rectal surgery and excluded transanal approaches such as TTSS and TaTME. This was done to improve comparability and isolate the effect of anastomotic configuration within abdominal-approach rectal resection. Nevertheless, several limitations deserve consideration. First, most included studies were retrospective, technical reports or small observational series, resulting in an overall low-to-moderate level of evidence. Second, important clinical variables including anastomotic height, tumor location, diverting stoma use and neoadjuvant therapy were inconsistently reported, precluding clinically meaningful subgroup analyses according to baseline risk. Third, aggregate event rates were derived from crude pooled data rather than formal meta-analytic methods and should therefore be interpreted as descriptive rather than comparative findings. Fourth, differences in surgical indications, patient selection, and the inclusion of intracorporeal reconstruction with transanal specimen extraction in some studies may have introduced additional confounding unrelated to the anastomotic configuration itself.

## Conclusions

This review provides a comprehensive technical classification of transabdominal strategies designed to eliminate or neutralize staple-line intersection. Techniques appear technically feasible and associated with low reported leak rates. Modified DST approaches may represent a pragmatic compromise between technical simplicity and optimization of the anastomotic configuration, while aSST remains technically demanding and currently supported by limited heterogeneous evidence. Prospective comparative studies are required to determine whether complete elimination of staple-line intersection translates into clinically meaningful reductions in anastomotic leakage.

## Data Availability

Datasets generated are available upon reasonable request.
